# Avoidance of wind farms by harbour seals is limited to pile driving activities

**DOI:** 10.1111/1365-2664.12678

**Published:** 2016-05-23

**Authors:** Debbie J.F. Russell, Gordon D. Hastie, David Thompson, Vincent M. Janik, Philip S. Hammond, Lindesay A.S. Scott‐Hayward, Jason Matthiopoulos, Esther L. Jones, Bernie J. McConnell

**Affiliations:** ^1^ Sea Mammal Research Unit University of St Andrews St Andrews Fife KY16 8LB UK; ^2^ Centre for Research into Ecological and Environmental Modelling University of St Andrews St Andrews Fife KY16 9LZ UK; ^3^ Institute of Biodiversity Animal Health, and Comparative Medicine University of Glasgow Graham Kerr Building Glasgow G12 8QQ UK

**Keywords:** Complex Region Spatial Smoother, disturbance, marine renewables, marine spatial planning, pinnipeds, renewable energy, Spatially Adaptive Local Smoothing Algorithm, spatially adaptive smoothing, underwater noise

## Abstract

1. As part of global efforts to reduce dependence on carbon‐based energy sources there has been a rapid increase in the installation of renewable energy devices. The installation and operation of these devices can result in conflicts with wildlife. In the marine environment, mammals may avoid wind farms that are under construction or operating. Such avoidance may lead to more time spent travelling or displacement from key habitats. A paucity of data on at‐sea movements of marine mammals around wind farms limits our understanding of the nature of their potential impacts.
2. Here, we present the results of a telemetry study on harbour seals *Phoca vitulina* in The Wash, south‐east England, an area where wind farms are being constructed using impact pile driving. We investigated whether seals avoid wind farms during operation, construction in its entirety, or during piling activity. The study was carried out using historical telemetry data collected prior to any wind farm development and telemetry data collected in 2012 during the construction of one wind farm and the operation of another.
3. Within an operational wind farm, there was a close‐to‐significant increase in seal usage compared to prior to wind farm development. However, the wind farm was at the edge of a large area of increased usage, so the presence of the wind farm was unlikely to be the cause.
4. There was no significant displacement during construction as a whole. However, during piling, seal usage (abundance) was significantly reduced up to 25 km from the piling activity; within 25 km of the centre of the wind farm, there was a 19 to 83% (95% confidence intervals) decrease in usage compared to during breaks in piling, equating to a mean estimated displacement of 440 individuals. This amounts to significant displacement starting from predicted received levels of between 166 and 178 dB re 1 μPa_(p‐p)_. Displacement was limited to piling activity; within 2 h of cessation of pile driving, seals were distributed as per the non‐piling scenario.
5. *Synthesis and applications*. Our spatial and temporal quantification of avoidance of wind farms by harbour seals is critical to reduce uncertainty and increase robustness in environmental impact assessments of future developments. Specifically, the results will allow policymakers to produce industry guidance on the likelihood of displacement of seals in response to pile driving; the relationship between sound levels and avoidance rates; and the duration of any avoidance, thus allowing far more accurate environmental assessments to be carried out during the consenting process. Further, our results can be used to inform mitigation strategies in terms of both the sound levels likely to cause displacement and what temporal patterns of piling would minimize the magnitude of the energetic impacts of displacement.

## Abstract 2

Our spatial and temporal quantification of avoidance of wind farms by harbour seals is critical to reduce uncertainty and increase robustness in environmental impact assessments of future developments. Specifically, the results will allow policymakers to produce industry guidance on the likelihood of displacement of seals in response to pile driving; the relationship between sound levels and avoidance rates; and the duration of any avoidance, thus allowing far more accurate environmental assessments to be carried out during the consenting process. Further, our results can be used to inform mitigation strategies in terms of both the sound levels likely to cause displacement and what temporal patterns of piling would minimize the magnitude of the energetic impacts of displacement.

## Introduction

Wind farms are increasingly being established offshore to avoid adverse public opinion and exploit more consistent wind patterns (Inger *et al*. 2009). In the north‐east Atlantic, there are currently 54 operational wind farms (2925 turbines), 70 more have been consented and applications for a further 86 have been submitted (OSPAR; http://www.ospar.org/data; downloaded 7 August 2015). Wind farms have the potential to impact the marine ecosystem during both their construction and operation. For animals, such as marine mammals, that are highly sensitive to underwater sound, it is during the construction phase that wind farms are predicted to have the greatest impact (Bailey, Brookes & Thompson 2014). Specifically, offshore wind turbine foundations are commonly installed using impact pile driving which produces intense impulse sounds under water (Madsen *et al*. 2006); these have the potential to elicit overt behavioural responses in marine mammals (Tougaard *et al*. 2009; Dähne *et al*. 2013).

Due to the inherent difficulties in observing marine mammals at sea, studies on noise‐induced displacement of seals have mostly focussed on either captive playback studies or counts at haulout sites. Captive studies of harbour and grey seals *Halichoerus grypus*, have demonstrated behavioural aversion to high‐level sounds (Kastelein *et al*. 2006; Götz & Janik 2010) including playbacks of pile driving (Kastelein *et al*. 2013). Numbers of grey and harbour seals at a local haulout site appeared to vary in response to nearby pile driving activities (Edrén *et al*. 2009) but the construction phase as a whole was not associated with changes in haulout abundance (Teilmann *et al*. 2006). However, it remains unclear whether seals exhibit any at‐sea avoidance of wind farms under construction. The magnitude of energetic consequences of any displacement will depend, *inter alia*, on the temporal and spatial scale of any displacement.

Potential impacts of operational wind farms on marine mammals also need to be considered when assessing the ecological impacts of wind farms; these may be more complex than those of construction and occur over a longer temporal period. Marine mammals could be displaced from existing wind farms either due to operational noise or because of disturbance by maintenance vessels (Tougaard, Henriksen & Miller 2009). In contrast, wind farms may also cause an increase in abundance of some species (Scheidat *et al*. 2011): restrictions on ship traffic may result in decreased disturbance, and the exclusion of some types of fishing may result in decreased bycatch and increased prey availability (Inger *et al*. 2009). Recent evidence shows that individual harbour seals use wind farms for foraging likely due to artificial reefs on the turbine foundations (Russell *et al*. 2014). However, a quantitative analysis of changes in harbour seal usage around operational wind farms has not been carried out.

**Figure 1:**
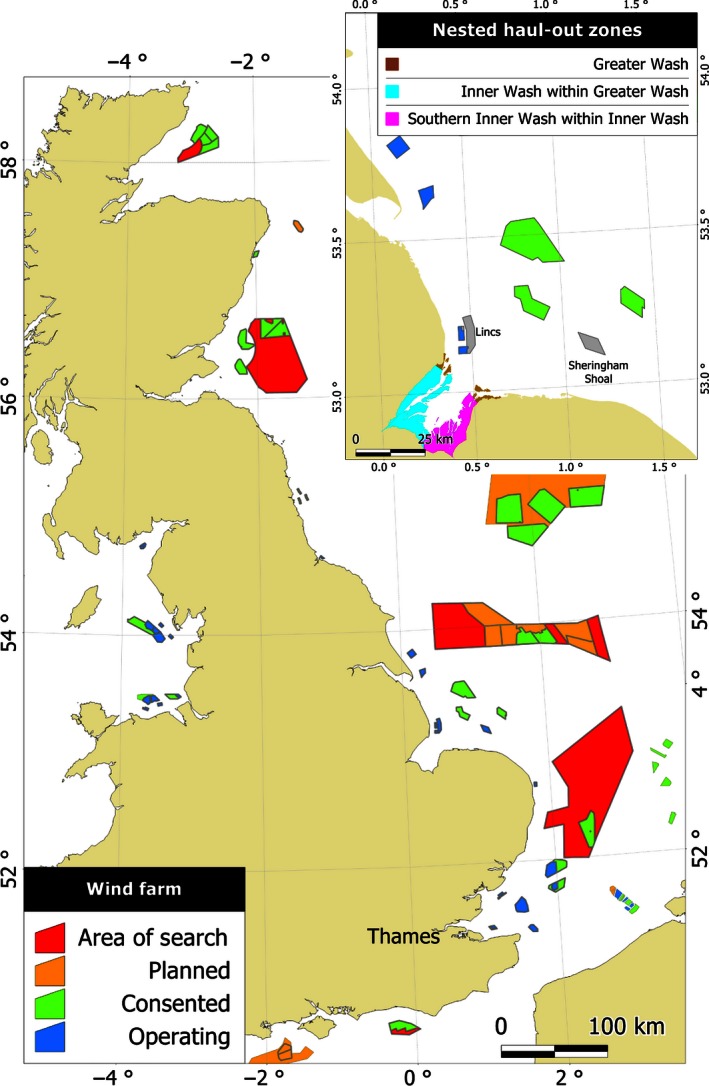
Wind farms at indicated stages of development as per Crown Estate (http://www.thecrownestate.co.uk/energy-and-infrastructure/downloads/maps-and-gis-data/) and OSPAR (http://www.ospar.org/data; downloaded 7 August 2015). The magnified box indicates the area of the study including the haulout zones and the wind farms considered in this study (shown in grey).

The paucity of information regarding the effects of wind farm construction and operation on harbour seal behaviour currently limits the predictions of environmental impacts of offshore wind farms (Madsen *et al*. 2006; Inger *et al*. 2009; Thompson *et al*. 2013b). Such information is required to inform the consenting process of offshore developments; in the European Union, harbour seals are listed as Annex II species of the European Habitats Council Directive (92/43/EEC) requiring EU member states to designate Special Areas of Conservation (SAC) for harbour seals. If developments have the potential to have a significant effect on the integrity of a SAC (Council of the European Communities 1992), an Appropriate Assessment is required. Developments may only be permitted if it is determined that the development, individually or in combination with other impacts, will not adversely affect the integrity of the site once any identified impacts have been mitigated against. Analytical frameworks have recently been developed to inform this process by predicting the impact of wind farm developments on the populations of species such as harbour seals (Thompson *et al*. 2013b; King *et al*. 2015). However, Thompson *et al*. (2013b) highlight that a key uncertainty in these frameworks is the extent to which predicted noise from wind farm construction may impact seal behaviour; there is an urgent requirement for information on sound levels that elicit displacement by seals, and the recovery times after any displacements (Thompson *et al*. 2013b). In the current study, we look to address the paucity of data on seal behaviour around wind farms. Specifically, we present data from animal‐borne tags deployed on harbour seals in a SAC in the southern North Sea. In this study, we use telemetry data to compare spatial usage prior to wind farm development and during the construction of one wind farm, and the partial operation of another (Fig. 1). As such, we quantify the changes in at‐sea harbour seal usage during (i) operational activities, (ii) construction as a whole and (iii) individual pile driving bouts.

## Materials and methods

### Study Site

Approximately 13% of the UK population of harbour seals regularly use intertidal sand banks in The Wash to haul out between foraging trips and to breed (Duck, Morris & Thompson 2014). Near the mouth of The Wash, four wind farms have been constructed (217 turbines) and three further wind farms (maximum of 471 turbines) have been consented (Fig. 1). Pile driving associated with the first two wind farms (Inner Dowsing and Lynn) occurred in 2007, and they began operating in 2008. Piling for Sheringham Shoal started in 2010 and finished in August 2011 when this wind farm became operational. By the end of this telemetry study, June 2012, Sheringham Shoal wind farm was partially operational with 30 of 88 turbines operating. Pile driving at the Lincs wind farm occurred between May 2011 and May 2012, and the farm started operating in August 2012.

Each year, harbour seals in The Wash are counted by aerial survey while they haul out during their August moult. These counts provide an index of population size that showed an increase from 1695 individuals in 2006 to 3372 in 2012 (Duck, Morris & Thompson 2014). The proportion of harbour seals hauled out during the moult surveys estimated from telemetry data is 0.72 (95% CIs: 0·54–0·88; Lonergan *et al*. 2013), resulting in a population estimate for The Wash in 2012 of 4683 individuals (median; 95% CIs: 3832–6244). The proportion of time seals spent at sea during our study period (January to May) was estimated to be 0·834 (Russell *et al*. 2015) resulting in an estimated 3906 individuals at sea at any one time.

### Telemetry Data

Seals were caught on or close to haulout sites using hand or seine nets. Telemetry tags were attached to the fur at the back of the neck using a fast‐setting two‐part epoxy adhesive or Loctite^®^ 422 Instant Adhesive. All seal handling and procedures were carried out under Home Office Licences 60/3303 and 60/4009. Capture and handling procedures are described in more detail in Sharples *et al*. (2012).

**Figure 2:**
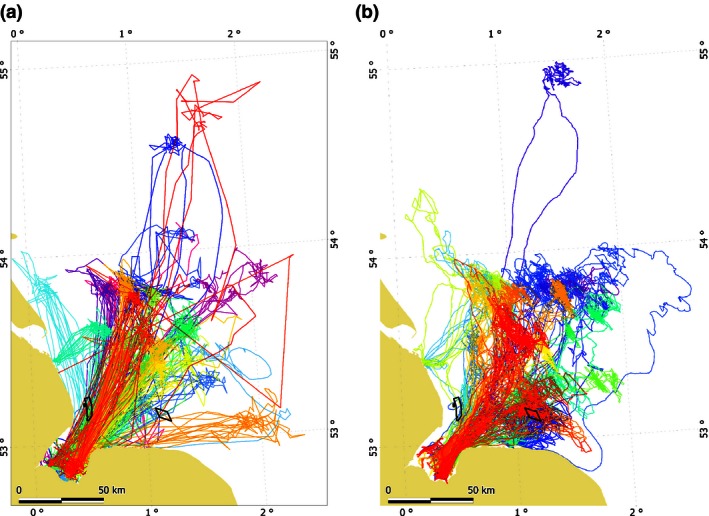
All telemetry tracks from two different sets of harbour seals using the Wash to haul out: historical ARGOS data (a, *n* = 25, years 2003–2006) and 2012 GPS data (b, *n* = 24). In each panel, each colour represents the track of a different individual. The Lincs (west) and the Sheringham Shoal (east) wind farms are outlined in black.

Prior to any wind farm construction in the vicinity of The Wash, a total of 24 ARGOS Satellite Relay Data Logger (SRDL) tags (Sharples *et al*. 2012) were deployed on harbour seals there in 2003, 2004 and 2005. In addition, of nine individuals tagged in 2006 in the Thames, over 150 km to the south of The Wash (Fig. 1), one male travelled to The Wash from where it performed multiple trips to sea. This resulted in locational data from 25 individuals (Sharples *et al*. 2012) (Fig. 2a) with tag durations of between 69 and 201 days. Haulout data were not transmitted for six of the individuals tagged in 2004, and thus, these individuals were excluded from further analysis, resulting in a sample size of 19 individuals. Locational data from ARGOS are subject to substantial location error, so a Kalman Filter was used to estimate locations as described in Jones *et al*. (2015). The median frequency of ARGOS locations for the 19 tags (10 females, nine males) considered in this study was seven per day.

In January 2012, 25 GPS phone tags were deployed in The Wash; 22 (12 females, 10 males) of which transmitted data for over 10 days and were therefore included in further analyses. We excluded data from one individual tagged in 2012 for which there was only two trips out with the Wash; one trip went much further than the other individuals and preliminary analysis revealed that inclusion of that trip would have resulted in a much larger accessible area and issues in model selection by cross‐validation (see Analysis). Also in January 2012, ten tags were deployed in the Thames; two of these individuals (one female and one male) travelled to The Wash from where they made multiple return trips. This resulted in a sample size of 23 individuals (tag duration between 19 and 172 days; Fig. 2b). At‐sea distribution during the breeding season from June to July is affected by breeding status (Thompson *et al*. 1994; Van Parijs *et al*. 1997), which was not known in this study. We therefore excluded data from this period, so our data terminated at the end of May. Locational data from GPS phone tags are of higher precision than data from ARGOS tags. Nonetheless, erroneous locations do occur and these were removed (Russell *et al*. 2015). The temporal resolution of the data from the GPS phone tags was higher than from the ARGOS tags; GPS tags gave a median of 77 locations per day. The tags also provided summarized behavioural data at a resolution of 6‐ and 2‐h periods for ARGOS and GPS tags, respectively.

### Data Preparation

We considered whether harbour seals demonstrated changes in usage around wind farms at two temporal scales. First, we compared the at‐sea distribution of harbour seals prior to any development (historical data 2003–2006) and in 2012 when one wind farm, Lincs, was under construction and another, Sheringham Shoal, was partially operational. Secondly, we compared the at‐sea distribution during periods of piling at Lincs, with non‐piling periods. Analyses were split to allow examination of the 2012 data at a higher temporal resolution (non‐piling vs. piling) than could be used when also considering the historical data (historical vs. 2012).

#### Piling data

Lincs wind farm developer (Centrica plc) provided data on pile driving which occurred throughout the 2012 tag deployment; 27 piles (of a total of 75) were installed requiring, on average, 2887 blows each. 70% of the piles were each driven within a 24‐h period, taking a mean of 5·85 h to install. However, there were a number of prolonged gaps during the installation of piles; the longest was 19 days. For individual piling events blow energy ranged from around 100 to 2000 kJ. Acoustic source levels were derived using a combination of the blow energy values and acoustic recordings made using an autonomous underwater recorder (see Hastie *et al*. 2015 for more details). We used sound pressure level (SPL) and sound exposure level (SEL) to relate changes in seal usage to sound exposure. SPL is a decibel value relative to a standard reference pressure of 1 μPa in water. The SEL takes the different duration of sounds into account and is a measure of the accumulated energy over a defined period (here 1 s). It is the integral of the squared acoustic pressure with respect to time, expressed as a level in dB over the defined period. The predicted maximum SPL at source at the maximum blow energy was 235 dB re 1 μPa_(p‐p)_ @ 1 m. For the purposes of this study, we assumed that single pulse sound exposure levels (SELs) were 24 dB lower than SPLs, resulting in a predicted maximum SEL at 1 m from the source of 211 dB re 1 μPa^2^ s^−1^ (Hastie *et al*. 2015). For each pile and 5 × 5 km grid cell in our study area (see Predictions), a series of range‐dependent acoustic propagation models were used to predict received SPLs and SELs at 5 m incremental water depths (Hastie *et al*. 2015) based on the maximum pile driving source level found in our study. Predicted received SPLs and SELs were averaged for each cell across the installation of all piles, to generate a mean received SPL and SEL in the part of the water column with the lowest and highest predicted level (Fig. S1 in Supporting Information). The analysis of change of usage was conducted by comparing usage on the scale of piling and non‐piling (see Analysis), and thus, averaging received levels across piles was required to allow us to relate changes in seal usage to minimum and maximum predicted received levels.

#### Historical vs. 2012

A temporal resolution of 6 h was dictated by the resolution of the historical ARGOS data. All locations from both the historical and 2012 data were linearly interpolated to produce one location in the middle of each 6‐h period. To enable comparisons between historical and 2012 data, historical data were restricted to the same seasonal extent as the 2012 data (January to May). With the exception of those tagged in the Thames, all individuals were tagged in The Southern Inner Wash, but they used other haulout zones (Fig. 1) to varying degrees. Harbour seals are effectively central place foragers, returning regularly to land, and thus, their distribution at sea is likely to be affected by the location of that central place (haulout site). To ensure an unbiased comparison between the historical and 2012 data, ideally only return trips (where the departure and destination haulout were the same) from The Southern Inner Wash would be included in our analyses. Low positional accuracy for the historical data meant that it was often impossible to pinpoint haulout sites to The Southern Inner Wash. Instead, return trips from a larger area, The Inner Wash, which includes The Southern Inner Wash (Fig. 1), were retained. The exclusion of trips from elsewhere in The Wash did not result in the loss of many data because 98% of haulout events in The Greater Wash were within The Inner Wash.

#### Non‐piling vs. piling

The tags deployed in 2012 provided data at a higher temporal resolution, so the location data were linearly interpolated to provide one location at the mid‐point of each 2‐h period. The higher spatial accuracy of the 2012 data meant that we could allocate haulouts more precisely so only return trips from The Southern Inner Wash were included in this analysis; 94% of haulout events in The Greater Wash were within The Southern Inner Wash. Periods were flagged as ‘piling’ if any piling activity was recorded within the period.

### Analyses

#### Use–availability design

The location of an individual is a reflection of both where it can go (accessibility) and where it chooses to be (preference; Matthiopoulos 2003). The maximum geodesic distance (shortest path at sea) of the return trips from the haulout zones (Fig. 1) were used to define the accessible area. For each presence point, within the accessible areas we generated a randomly positioned pseudo‐absence (historical vs. 2012, *n* = 12 239; non‐piling vs. piling, *n* = 6744). These absence data can be thought of as representative samples of points from the region of space that is accessible to the seals, and therefore as a means of communicating to a model the contrast between the space actually used by the seals and the space that is broadly available to them in their environment (Beyer *et al*. 2010). The distribution was modelled as a binomial process (0 as absence and 1 as presence) as a function of a two‐dimensional smooth of longitude and latitude.

#### Model details

For both analyses (historical vs. 2012, and non‐piling vs. piling) we used a Complex Region Spatial Smoother (CReSS) with a Spatially Adaptive Local Smoothing Algorithm (SALSA) and cross‐validation for model selection for the location and number of knots, respectively. The CReSS smooth employs a local radial exponential basis function whose effective region of influence can be varied to be locally or globally acting (Scott‐Hayward *et al*. 2014). It allows specification of geodesic distances between all points and knots, thus taking into account complex coastlines such as The Wash. SALSA was originally developed for one‐dimensional smoothing (Walker *et al*. 2011) and recently adapted for CReSS two‐dimensional smooths specifically to address questions of the impact of marine renewable developments on animal distributions (Scott‐Hayward *et al*. 2013). These tools have some advantages over generalized additive models that are implemented in R library mgcv (Wood 2011), which can also be used to describe distributions, because within a smooth term they simultaneously allow both adaptive smoothing and the use of geodesic distances which reduces edge effects (see Appendix S1).

#### Model selection

The model incorporating a separate smooth for each temporal factor level was considered as the final model; that is, model selection was not used to decide whether there should be a separate smooth for each period (historical and 2012, or non‐piling and piling). This is because our aim was not to determine whether the distribution of seals across The Wash was better explained using one smooth per scenario or one overall smooth, but to determine whether there was a significant change in seal usage in relation to the wind farms. Thus, model selection was conducted to choose the most appropriate locations and numbers of knots for each smooth (see Appendix S2).

Once the optimal model was selected, it was rerun in a generalized estimating equation (GEE; Hardin & Hilbe 2002) framework. This allowed robust estimation of the precision associated with the spatial predictions because by using the independent working correlation structure we accounted for any residual autocorrelation within defined panels of data (Pirotta *et al*. 2011). GEEs have been previously used with telemetry data in a use–availability design (Bailey, Hammond & Thompson 2014) for which pseudo‐absences and presences were combined in individual‐specific panels. Here, we used separate panels for presences and absences to avoid underestimating the autocorrelation within the presences of an individual. A separate panel was used for the presences relating to each individual. Each pseudo‐absence was assumed to be independent and thus was included in a separate panel.

#### Non‐piling vs. piling: time to redistribute

If harbour seals were displaced when piling started their distribution during piling would have taken time to become realized as their maximum travel speed is about 2 m s^−1^ (McClintock *et al*. 2013). Furthermore, we wanted to determine how long, once piling had ceased, it took for them to redistribute back to the non‐piling scenario. Thus, we investigated, using model selection, whether seal distribution was best explained when the initial (first, second, etc.) piling and non‐piling periods were assigned to non‐piling and piling, respectively. We conducted model selection based on reassigning of periods until increasing the number of periods reassigned lowered model fit (as measured by cross‐validation). Comparisons of model fit required equal sample sizes but since the reassigned periods occurred during transition between the two distributions, these periods were excluded for final model fitting and prediction.

#### Predictions

Due to the use–availability design of the study, predictions of abundance were based on the exponential of the linear predictions from the logistic model (Beyer *et al*. 2010). For the area available to the seals, we predicted the seal usage and differences therein, on a 5 × 5 km grid. A parametric bootstrap from the GEE model was used to calculate 95% confidence intervals (CIs) for both the predicted usage (percentage of the at‐sea population) and predicted change in usage (historical to 2012 or non‐piling to piling). We also predicted how the change in usage between non‐piling and piling periods was related to distance from the middle of the Lincs wind farm and the received SPLs and SELs (averaged across all installations) in the part of the water column with the lowest and highest predicted levels. Using the estimated population of The Wash in 2012 which would have been at sea at any one time (3906; see Study Site), and the predicted changes in percentage usage, we approximated the change in the number of individuals within areas of interest.

#### Software

All data preparation and analysis were carried out using R (R Core Team 2012) within packages fields (Furrer, Nychka & Sain 2012), geepack (Højsgaard, Halekoh & Yan 2006), rgdal (Keitt *et al*. 2013), sp (Pebesma & Bivand 2005), splancs (Rowlingson *et al*. 2013) and MRSea (Scott‐Hayward *et al*. 2013).

## Results

### Historical vs. 2012

**Figure 3:**
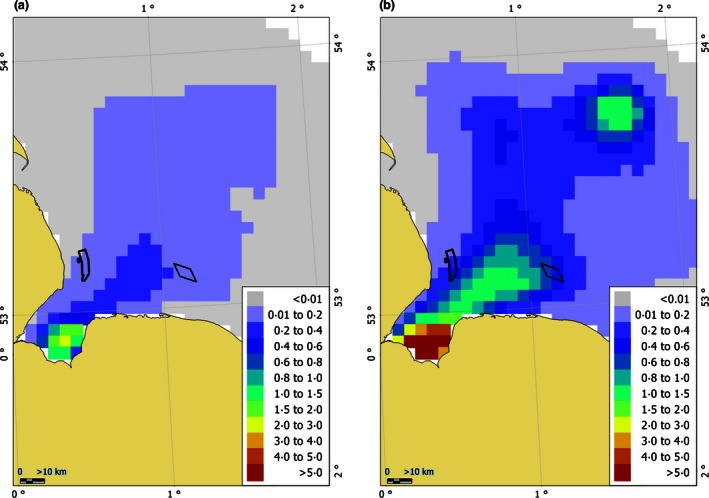
The predicted distribution of harbour seals on return trips from the Inner Wash at a 5‐km resolution in 2012. The metric is the percentage of the at‐sea population with the lower (a) and upper (b) 95% confidence limits per cell shown. The outline of Lincs (west) and Sheringham Shoal (east) wind farms is also shown.

Seven of the individuals tagged in The Wash and one in the Thames entered Sheringham Shoal which was partially operational; five did so on multiple occasions. The model selected by cross‐validation (14 knots) revealed that there was a close‐to‐significant (at the 5% level) increase in seal usage of Sheringham Shoal in 2012 compared to prior to its existence. Between 0.01 and 1.16% of seals occupied the cells which now encompass Sheringham Shoal before it was built, compared to 0.54 and 2.82% in 2012 when it was operational. Within the 5‐km cells which now encompass Lincs, there was a significant increase in seal usage in 2012 compared to historical data. Prior to wind farm construction, at any one time between 0·05 and 0·39% (95% CIs) of seals at sea were within the cells encompassing Lincs (Fig. S2) compared to between 0·28 and 1·89% in 2012 (Fig. 3). If we consider the estimated population size in 2012, the mean estimated change historically to 2012 would be equivalent to an increase of approximately 50 and 25 individuals at any one time in Sheringham Shoal and Lincs, respectively.

### Non‐Piling vs. Piling

**Figure 4:**
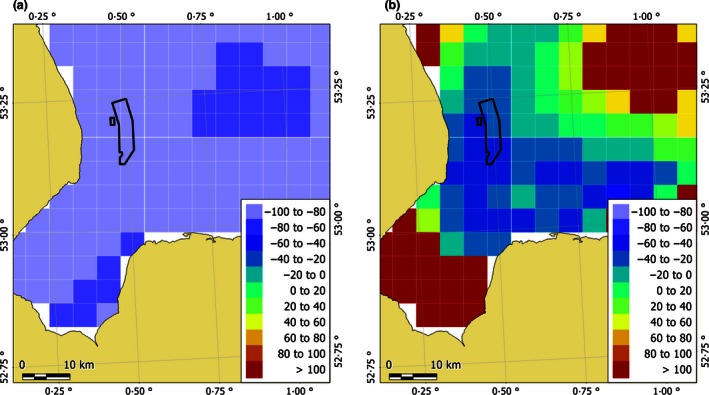
The change between the non‐piling and piling at‐sea distributions. The metric is the percentage change in the at‐sea population; cool colours indicate decreased usage and warm colours indicate an increase in usage with the lower (a) and upper (b) 95% confidence limits per cell shown. The cells encompassing Lincs wind farm (outline shown in black) show a percentage decrease in usage of 20–100%.

**Figure 5:**
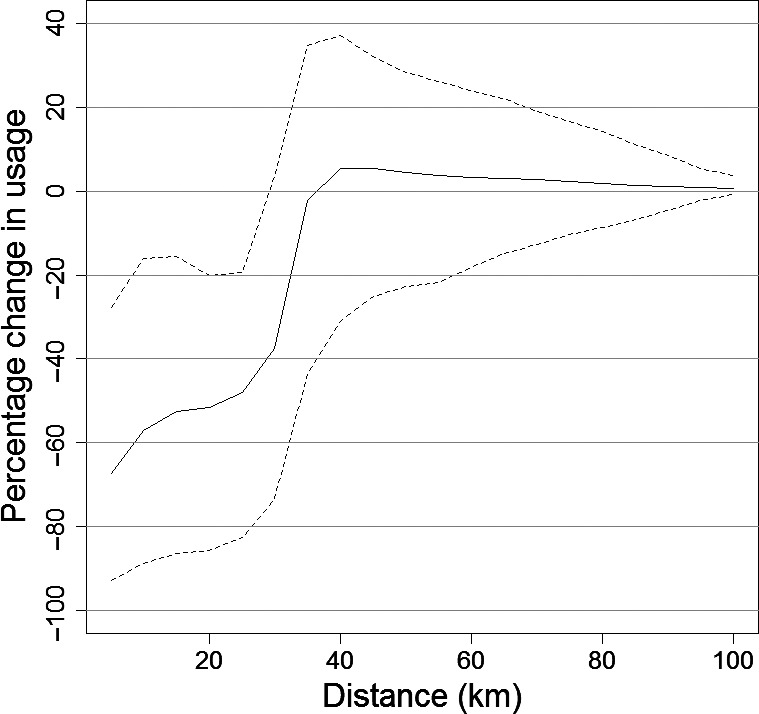
The predicted percentage change in usage during piling compared to non‐piling with regard to distance from the centre of Lincs wind farm. The dashed lines show the 95% confidence intervals.

**Figure 6:**
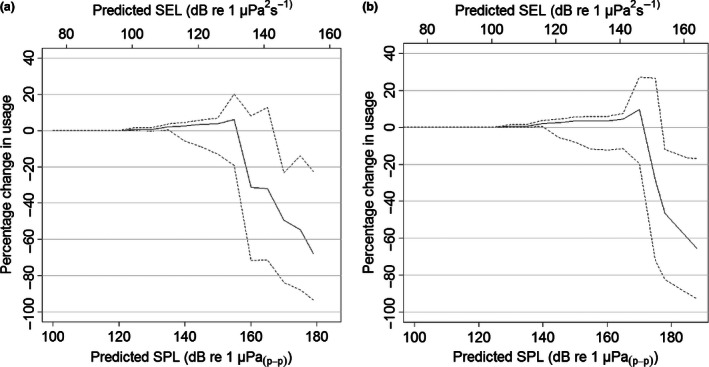
During piling, the predicted percentage change in usage compared to non‐piling at the predicted received sound pressure levels (SPLs) and sound exposure levels (SELs) from the pulse with the highest source level (averaged across installations) for parts of the water column with the lowest (a) and highest (b) received SPLs and SELs. SEL was calculated to be 24 dB lower than SPL. The dashed lines show the 95% confidence intervals.

Using the model selected by cross‐validation (21 knots), we found that in an area extending 25 km from the centre of the wind farm there was a significant percentage decrease in seal usage during piling (Fig. 4) of between 19 and 83% (95% CIs), equating to an estimate of the displacement of approximately 440 individuals during piling. Within 5 km of piling, the percentage decrease in usage was between 27 and 93%. The percentage decrease in usage did not have a linear relationship with distance from the wind farm (Fig. 5, Figs S3 and S4). When compared to the predicted acoustic received levels, usage significantly decreased during piling at predicted received SPLs (averaged across all installations) from between 166 and 178 dB re 1 μPa_(p‐p)_ (Fig. 6) and at SELs from between 142 and 151 dB re 1 μPa^2^ s^−1^ in the part of the water column with the lowest and highest predicted levels. Using model selection we found that the distribution was best explained by reassigning the first non‐piling and piling periods to piling and non‐piling, respectively. In other words, it took 2 h for the distribution to be realized in response to piling and also for the distribution to return to normal once piling had ceased.

## Discussion

The results of this study provide the first measurements of the at‐sea distribution of seals in relation to wind farm construction and operation. We found no evidence that harbour seals were displaced from an operational wind farm; there was a near significant increase in usage of the area encompassing Sheringham Shoal compared to prior to construction. However, the wind farm was at the edge of an area of increased usage so the presence of the wind farm was unlikely to be the cause (Fig. 3 and Fig. S2). One individual concentrated its apparent foraging effort at the foundations of the individual turbines (Russell *et al*. 2014). Whether the wind farm will cause an overall increase in usage in time remains to be seen and is likely to depend on whether any localized increases in prey availability are sustained (Russell *et al*. 2014). We also found no evidence of displacement during the construction period as a whole; there was significantly more usage within the grid cells encompassing Lincs during construction than historically. However, there was a marked change in usage across The Wash from historically to 2012, so the observed changes in usage were likely due to other extrinsic factors, such as changes in prey distribution or increased competition for prey resulting from the increasing local populations of both harbour and grey seals, rather than the construction of the wind farm.

Our results showed that there was a significant displacement of seals during periods when pile driving was taking place, up to 25 km from the centre of the wind farm (Fig. 5). The distance that significant displacement extended to, appears similar to that recorded previously for harbour porpoises in response to pile driving (Tougaard *et al*. 2009). We predicted that harbour seals were displaced at SPLs of between 166 and 178 dB re 1 μPa_(p‐p),_ and at SELs of between 142 and 151 dB re 1 μPa^2^ s^−1^. A recent study of harbour porpoise responses to similar sounds (seismic airgun pulses) showed that relative density of porpoises decreased within 10 km of the survey vessel; SPLs in the region 5–10 km from source were similar to the levels reported in the current study (165 to 172 dB re 1 μPa_(p‐p)_ and 145–151 dB re 1 μPa^2^ s^−1^; Thompson *et al*. 2013a)_._ Additional work, based on individual responses to pile driving sound, is required to fully understand how received sound levels influence displacement, and how this may vary with location and behavioural context. For example, seals in our study area are likely to have been exposed previously to pile driving and it seems likely that seals naïve to the signals may exhibit different responses. The probability of an individual exhibiting a behavioural response to piling through avoidance of an area is likely to be affected not only by perceived sound levels but also by a range of internal factors that we were unable to incorporate (such as sex, behavioural state, hunger level, need to haul out, and reproductive status), as well as external factors (such as availability of prey; Götz & Janik 2010; Goldbogen *et al*. 2013). Although there was a significant decrease in usage extending across the entrance to The Wash, individuals continued to travel in and out of The Wash during piling (within 20 km of the wind farm; Fig. 3 in Hastie *et al*. 2015) suggesting that the motivation to forage offshore and haul out could outweigh the deterrence caused by piling. Such motivation may be partly responsible for the short recovery time observed in this study.

In terms of population consequences in our study area, there is no evidence of a negative effect on population growth caused by wind farm construction. Encompassing substantial variation within and between years, the population (moult and breeding counts) of The Wash (Duck, Morris & Thompson 2014) continued to increase throughout the construction of the wind farms, even at the haulout sites in The North‐East Wash which were closest to the construction. In fact, the rate of increase in The Wash (95% CIs: 10–13% per annum; Duck, Morris & Thompson 2014) is close to the theoretical maximum for this species (Härkönen, Harding & Heide‐Jørgensen 2002). However, some proposed wind farm sites (e.g. off the east coast of Scotland) are in areas of decreasing harbour seal populations (Duck, Morris & Thompson 2014), where any energetic impacts of avoidance during piling bouts may impose an additional stress on already compromised populations (Thompson *et al*. 2013b). Furthermore, the area encompassing the Lincs wind farm was not an area of high use, rather seals pass near it as they transit to and from foraging areas. The energetic costs of displacement from a key foraging area may be greater; it could result in reduced foraging opportunities or increased foraging competition in some areas. Nonetheless, it may prove beneficial for an individual to avoid areas during pile driving; the auditory system of marine mammals is likely to be vulnerable to damage from intensive sounds such as those produced in pile driving (Finneran *et al*. 2000, 2002) and a reduction in sound exposure through avoidance may reduce the risks of auditory damage. Despite evidence of avoidance, the seals in this study were still predicted to receive relatively high cumulative sound exposure levels (Hastie *et al*. 2015).

The results of our study have important implications for regulators assessing the environmental impacts of offshore wind developments in the planning consent process. Currently, the most common assessment approach is to carry out a quantitative prediction of numbers of individuals likely to be displaced as a result of wind farm pile driving, and of the spatial and temporal extents of this displacement. These predicted levels of displacement are then assessed against legislative requirements for the particular species and population in order to inform consenting. However, previous assessments have been constrained by the absence of data on behavioural responses of harbour seals to known levels of pulsed noise such as piling. For example, Thompson *et al*. (2013b) made conservative assumptions about the time that it takes seals to return to impacted areas, relying on information from harbour porpoises. The recovery time found for seals here (within 2 h after piling) is much shorter than found for harbour porpoises at a similar development (2–3 days; Brandt *et al*. 2011) and suggests that environmental assessments should focus on the potential impacts on seals of short‐term displacement (during piling) rather than displacement during construction as a whole. In terms of the spatial extent of avoidance, here it was limited to 25 km; however, differences in pile characteristics, and the effects of bathymetry on sound propagation, means that the displacement distance could vary significantly between sites (Madsen *et al*. 2006). Nevertheless, these results provide a clear pathway for regulators to produce guidance for industry on the likelihood of displacement of seals in response to pile driving; the relationships between sound levels and avoidance rates; and the duration of any avoidance. Such guidance should allow far more accurate environmental assessments to be carried out as part of the wind farm consenting process.

The results of this study also provide a clear avenue for spatial planning and the development of mitigation methods to allow wind farms to be developed in an environmentally sound manner. If displacement is restricted to periods when piling occurred, the temporal extent of any foraging disruption and scale of additional travel will be limited. Thus, the energetic costs of disturbance during construction (Madsen *et al*. 2006) may be relatively discrete. However, this highlights the importance of breaks in piling to allow seals to forage and travel unhindered. Such considerations are especially important in areas where multiple wind farms are due for development in parallel; the temporal and spatial aspects of displacement may be interactive and consideration of whether or not pile driving is carried out at different sites concurrently may prove important. Methods of mitigation, such as bubble curtains to reduce sound levels at source may prove to be effective in reducing displacement distances; there was a strong relationship between levels of displacement and predicted received levels. In terms of spatial planning, regulators need to consider the importance of the motivation for seals to forage and haul out; despite being deterred, if there is no other route available seals appear to be willing to continue to move through areas relatively close to pile driving to transit to and from haulout sites, potentially increasing the risks associated with being exposed to high levels of sound.

In summary, this study has shown that seals did not avoid an operational wind farm but that there was significant displacement of seals when pile driving was taking place as part of the construction of a wind farm; this recovered to pre‐piling levels within 2 h of the cessation of piling. The biological consequences of displacement remain poorly understood, and to understand the population‐level impacts of wind farms, the long‐term impacts on individual fitness, fecundity and survival need to be quantified.

## Supporting information


**Appendix S1.** Comparison with mgcv.
**Appendix S2.** Model Selection.
**Fig. S1.** The predicted received levels (dB re 1 μPa_(p‐p)_) during piling.
**Fig. S2.** The predicted historic distribution of harbour seals on return trips from the Inner Wash.
**Fig. S3.** The predicted distribution of harbour seals on return trips from The Southern Inner Wash during breaks in piling in 2012.
**Fig. S4.** The predicted distribution of harbour seals on return trips from The Southern Inner Wash during piling in 2012.
