# Supplementary material for: Avoidance of wind farms by harbour seals is limited to pile driving activities
Source: J Appl Ecol. 2016 May 23;53(6):1642–52. doi: 10.1111/1365-2664.12678 (PMC5111737; doi:10.1111/1365-2664.12678)
Supplement: Supplementary file 1 — Appendix S1. Comparison with mgcv. Appendix S2. Model Selection. Fig. S1. The predicted received levels (dB re 1 μPa(p‐p)) during piling. Fig. S2. The predicted historic distribution of harbour seals on return trips from the Inner Wash. Fig. S3. The predicted distribution of harbour seals on return trips from The Southern Inner Wash during breaks in piling in 2012. Fig. S4. The predicted distribution of harbour seals on return trips from The Southern Inner Wash during piling in 2012. [file JPE-53-1642-s001.docx]

Avoidance of windfarms by harbour seals is limited to pile driving activities

Supporting Information

Appendix S1. Comparison with mgcv

Generalised Additive Models (GAMs) within the mgcv library in R (Wood 2011) are often used to investigate spatial distributions of animals (e.g. Aarts *et al.* 2008). However, in this study they were deemed inappropriate for three reasons. First, due to our use availability design, our study area was large and in mgcv, by default, knots are placed evenly throughout the data in a smooth term. Such implementation would be inappropriate because the majority of the surface would be dominated by zeros. This would require a flat surface for much of the range and then a flexible surface for the range which contained both presences and absences. Due to the presence of a single smoothing parameter for each smooth term in mgcv, such varying flexibility across a surface is difficult to achieve. Second, in initial tests, we found substantial edge effects, possibly due to the complex nature of the area boundary (coastline). Although, both these issues can be addressed in mgcv using adaptive smoothing and soap-film smoothers, respectively, they cannot yet be used jointly in a single smooth term. Finally the structure of the data presented implementation difficulties: the data consist of serially correlated presence data and independent pseudo-absence data which do not fit into the auto-regressive serial correlation structures available in mgcv. A simulation exercise deemed CReSS and SALSA methods in a GAM-GEE framework to be the most appropriate, over mgcv GAMs and Generalised Additive Mixed Models, for environmental impact assessment data (Mackenzie *et al.* 2013).

Appendix S2. Model Selection

The following three part process was used to define 350 candidate knots for both analyses (historical vs. 2012 and non-piling vs. piling). (1) A 1 km grid was generated to cover the entire study area (2) Only grid points that were the nearest grid point to a data point (presence or pseudo-absence) were retained (3) A space filling design was used to evenly place 50 candidate knots throughout the pseudo-absences and 300 candidates throughout the presences, respectively. For the fine scale spatial variation in abundance to be accurately modelled, the majority of candidate knots were apportioned using the distribution of the presence data. However, it was important that candidate knots covered areas of absence to allow abundances for these areas to be appropriately estimated.

For each run of the model (24 runs), each with a specified number of knots (between 2 and 25), the position of the knots (of the 350 candidate knots) chosen by SALSA was that which minimised AIC. However, five-fold cross validation, which negates any issues of residual autocorrelation, was used to select the optimal model from the 24 candidate models. Each fold contained the data from 20% of the individuals; thus data from 80% of the individuals were used to fit the model and 20% to test it. Model selection by cross validation was conducted based on predictive performance of each model: the coefficient of the Spearman rank correlation between the binned predicted probabilities and the observed area-adjusted frequency of presences in the data (RSF score; Boyce *et al.* 2002; Wiens *et al.* 2008). The predicted probabilities bins were normalised equal-area bins with moving window averaging, as recommended by Wiens *et al.* (2008).

REFERENCES

Aarts, G., MacKenzie, M., Mcconnell, B.J., Fedak, M.A. & Matthiopoulos, J. (2008) Estimating space-use and habitat preference from wildlife telemetry data. *Ecography*, **31**, 140–160.

Beyer, H.L., Haydon, D.T., Morales, J.M., Frair, J.L., Hebblewhite, M., Mitchell, M. & Matthiopoulos, J. (2010) The interpretation of habitat preference metrics under use-availability designs. *Philosophical transactions of the Royal Society of London. Series B, Biological sciences*, **365**, 2245–54.

Boyce, S.M., Vernier, P.R., Nielsen, S.E. & Schmiegelow, K.A. (2002) Evaluating resource selection functins. *Ecological modelling*, **157**, 281–300.

Mackenzie, M.L., Oedekoven, C.S., Skov, H., Humphreys, E. & Rexstad, E. (2013) *Statistical Modelling of Seabird and Cetacean Data: Guidance Document. University of St. Andrews Contract for Marine Scotland; SB9 (CR/2012/05).*

Wiens, T.S., Dale, B.C., Boyce, M.S. & Kershaw, G.P. (2008) Three way k-fold cross-validation of resource selection functions. *Ecological Modelling*, **212**, 244–255.

Wood, S.N. (2011) Fast stable restricted maximum likelihood and marginal likelihood estimation of semiparametric generalized linear models. *Journal of the Royal Statistical Society. Series B: Statistical Methodology*, **73**, 3–36.

| (a) | 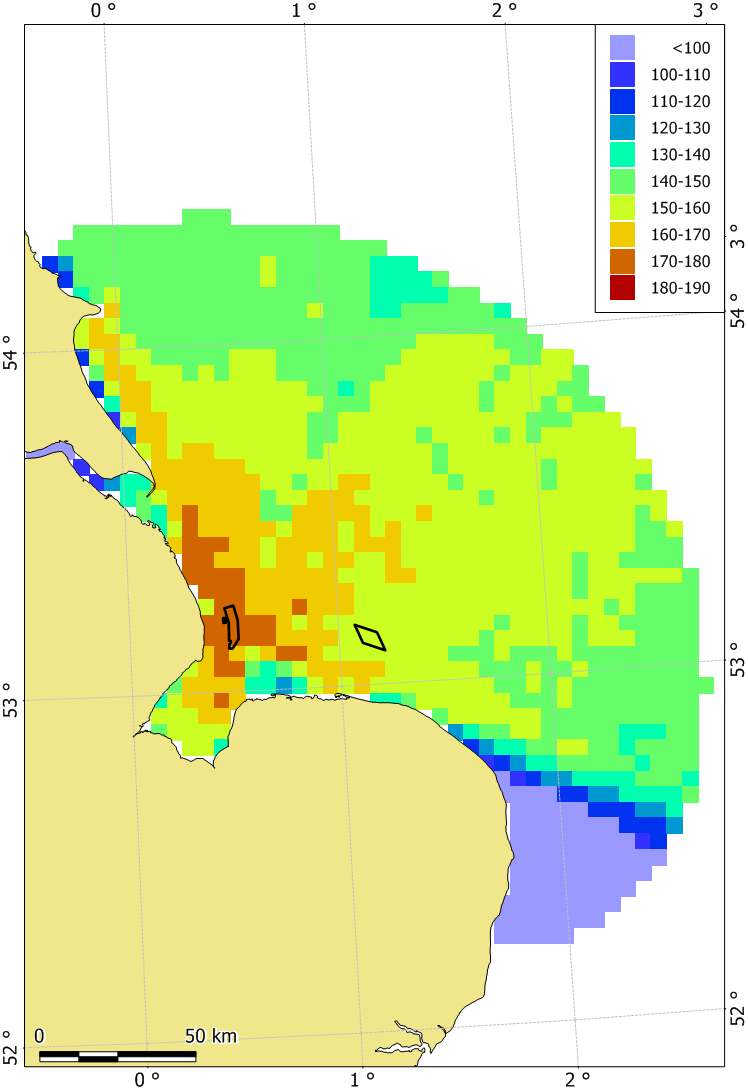 | (b) | 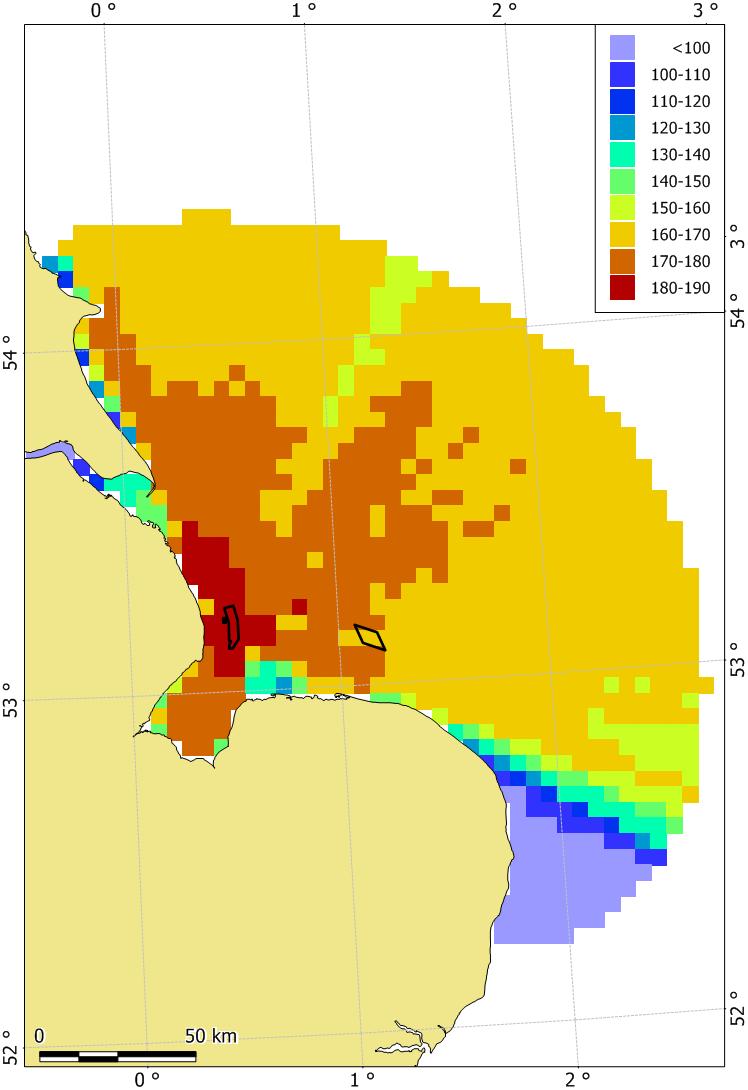 |
| --- | --- | --- | --- |

Figure S1. The predicted received levels (dB re 1µPa_(p-p)_) in the quietest (a) and loudest (b) part of the water column. The maximum peak to peak sound level was used to predict a received level for each cell and each water depth. The minimum and maximum received levels were then averaged across piles. The outline of Lincs (west) and Sheringham Shoal (east) windfarms are also shown.

| (a) | 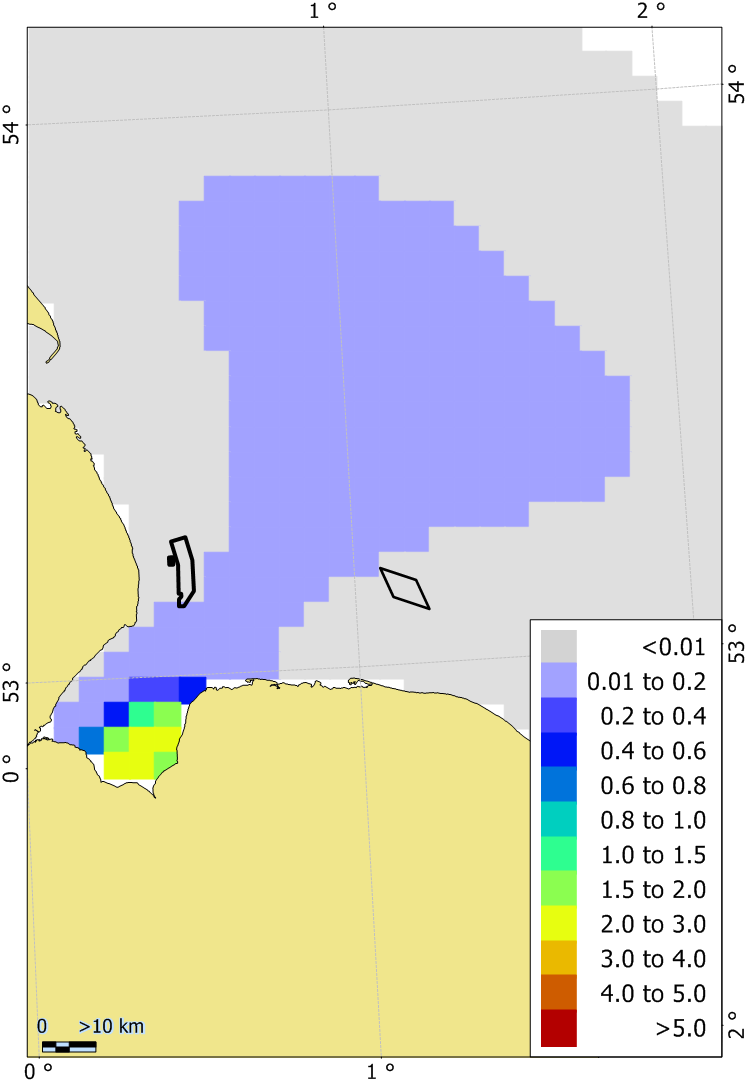 | (b) | 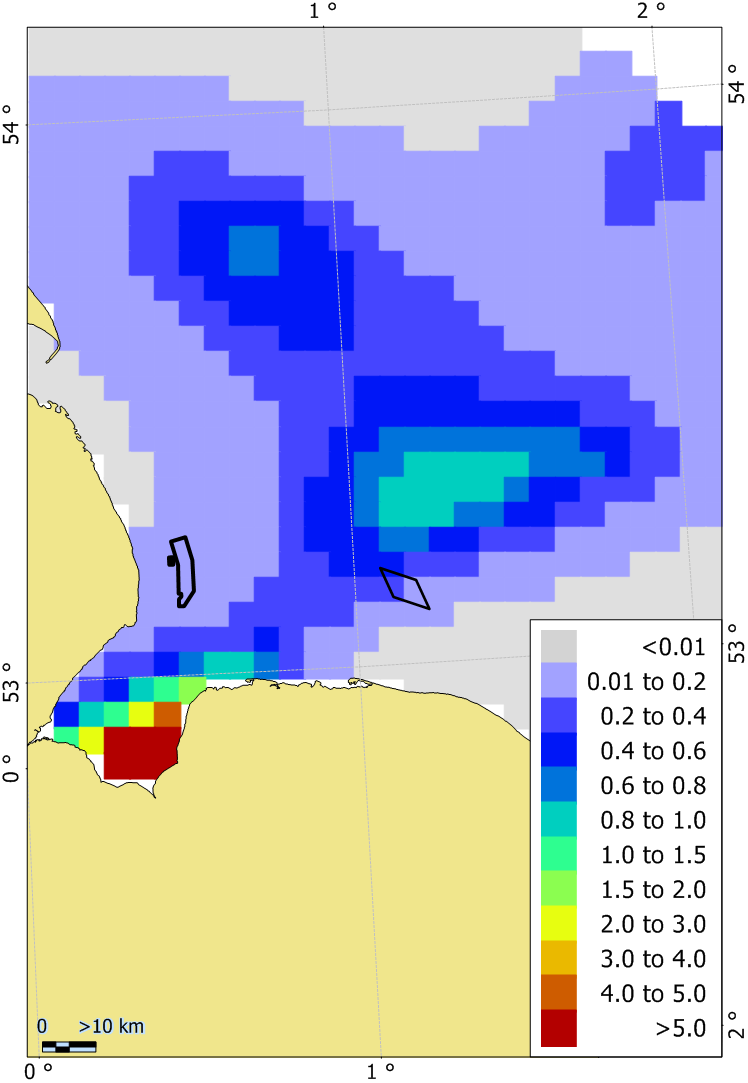 |
| --- | --- | --- | --- |

Figure S2. The predicted historic distribution of harbour seals on return trips from the Inner Wash on a 5 km resolution. The metric is the percentage of the at-sea population with the lower (a) and upper (b) 95% confidence limits per cell shown. The outline of Lincs (west) and Sheringham Shoal (east) windfarms are also shown.

| (a) | 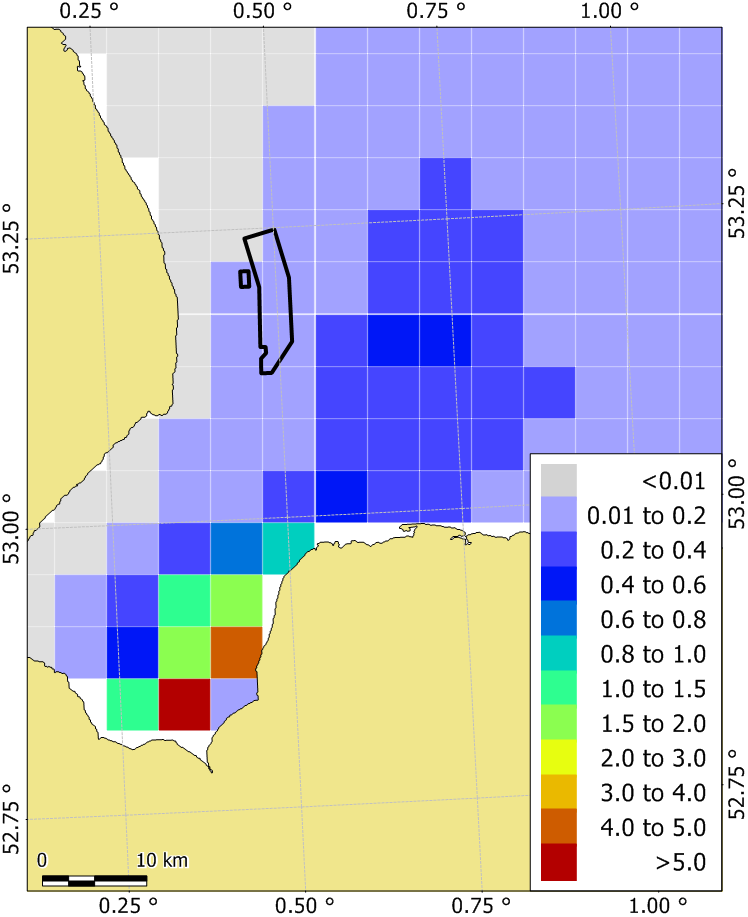 | (b) | 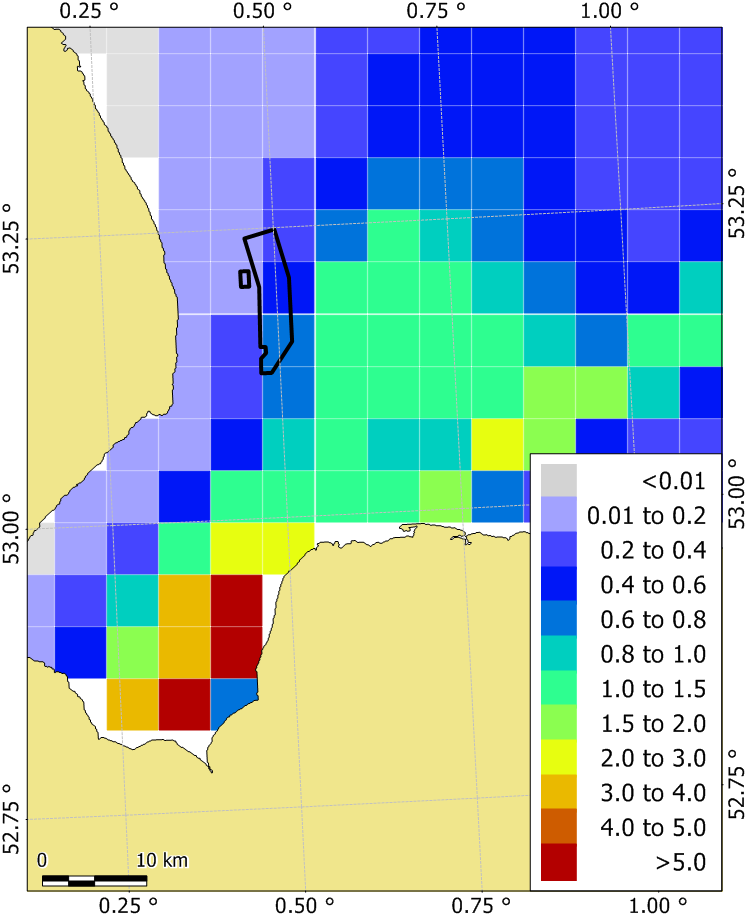 |
| --- | --- | --- | --- |

Figure S3. The predicted distribution of harbour seals on return trips from The Southern Inner Wash on a 5 km resolution during breaks in piling in 2012. The metric is the percentage of the at-sea population with the lower (a) and upper (b) 95% confidence limits per cell shown. The outline of Lincs windfarm is also shown.

| (a) | 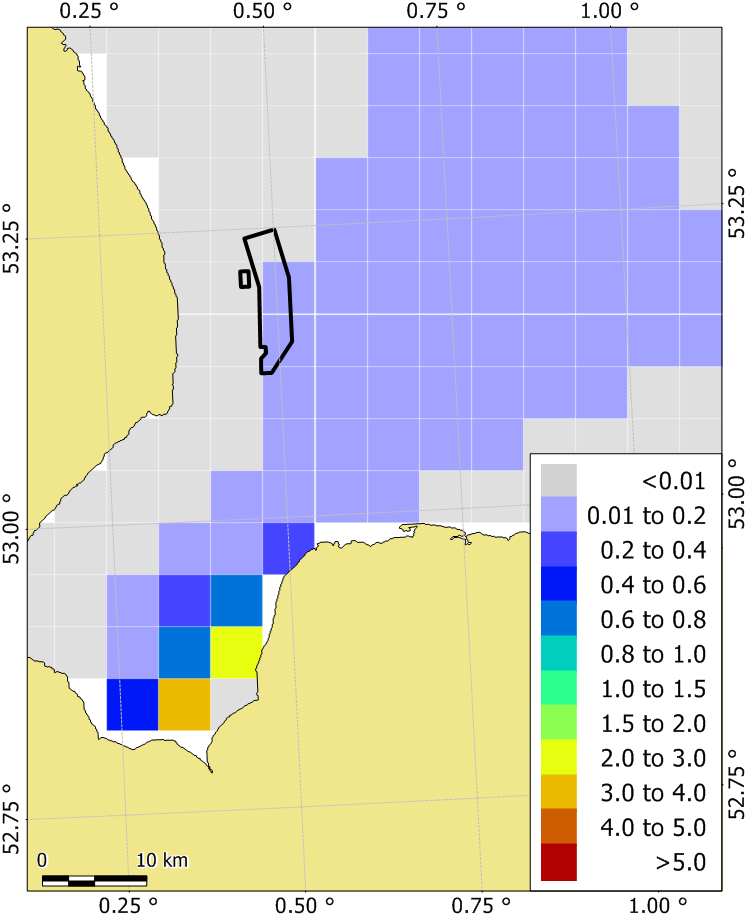 | (b) | 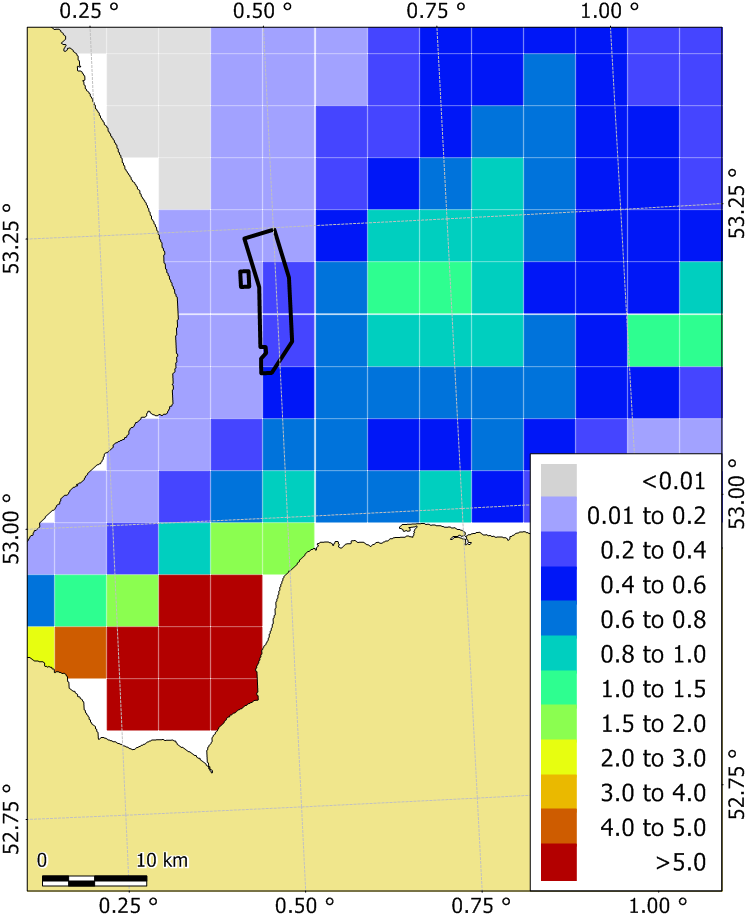 |
| --- | --- | --- | --- |

Figure S4. The predicted distribution of harbour seals on return trips from The Southern Inner Wash on a 5 km resolution during piling in 2012. The metric is the percentage of the at-sea population with the lower (a) and upper (b) 95% confidence limits per cell shown. The outline of Lincs windfarm is also shown.
